# The relationship between childhood trauma and romantic relationship satisfaction: the role of attachment and social support

**DOI:** 10.3389/fpsyt.2024.1519699

**Published:** 2025-01-22

**Authors:** Lijuan Quan, Kun Zhang, Haiyan Chen

**Affiliations:** School of Educational Science, Anhui Normal University, Anhui, Wuhu, China

**Keywords:** childhood trauma, romantic relationship satisfaction, attachment, social support, college students

## Abstract

**Objective:**

To investigate the impact of childhood trauma on romantic relationship satisfaction among college students, focusing on the mediating role of attachment and the moderating role of social support.

**Methods:**

A total of 1,404 college students from Wuhu, Anqing, Chaohu, Bengbu, Fuyang, and Wuhan participated in this study. Participants completed a series of self-report questionnaires, including the Childhood Trauma Questionnaire - Short Form (CTQ-SF), the Adult Attachment Scale (AAS), the Perceived Social Support Scale (PSSS), and the Romantic Relationship Satisfaction Scale (RRSS). Demographic variables such as grade were collected to control for potential confounding factors. Statistical analyses included correlation analyses, regression models, and moderated mediation analyses using PROCESS Macro.

**Results:**

Childhood trauma negatively predicted romantic relationship satisfaction both directly (*β* = -0.06, *t* = -2.11, *p* < 0.05) and indirectly through attachment (*β* = -0.07, *t* = -2.59, *p* < 0.05). Social support moderated the relationship between childhood trauma and attachment, with the effect of childhood trauma on attachment strengthening as social support increased (low: *t* = 2.18, *p* = 0.03; high: *t* = 4.37, *p* < 0.001). However, social support did not significantly moderate the direct effect of childhood trauma on romantic relationship satisfaction.

**Discussion:**

Attachment mediated the relationship between childhood trauma and romantic relationship satisfaction, while social support moderated the relationship between childhood trauma and attachment. These findings suggest that interventions should focus on improving attachment styles and strengthening social support to mitigate the negative effects of childhood trauma on romantic relationships.

## 1 Introduction

Humans, as social beings, have a basic need to engage in social interactions and build interpersonal relationships. Romantic relationships are an important part of individuals’ daily social networks (1). A satisfying romantic relationship can enhance trust and happiness between partners (2). However, when a relationship ends, individuals often experience significant distress (3). Romantic relationship satisfaction refers to an individual’s evaluation of their romantic (or marital) relationship and their subjective satisfaction with positive emotions such as happiness and pleasure (4). Previous research has shown that individuals with high romantic relationship satisfaction tend to have fewer depressive tendencies and experience less impact from negative life events (5, 6). Individuals with higher relationship satisfaction are more likely to maintain a long-term relationship, and this often results in a greater sense of self-worth and efficacy (7). In contrast, low satisfaction in romantic relationships may negatively impact academic performance and quality of life and is associated with an increased risk of depression (8). The age group of 18-25 is crucial for personality development in Erikson’s stages, where the main task is to develop intimacy and avoid loneliness. College students fall within this stage, making romantic relationship satisfaction especially significant for them.

Research has indicated that childhood trauma can have negative effects on individuals’ psychology, physiology, and behavior. Those who have experienced childhood trauma are more likely to evoke negative emotions, such as anxiety, depression, and anger (9–11). Studies have also shown that abuse during childhood may have harmful effects on mental health that persist into adulthood (12, 13). Individuals who experienced sexual and/or physical abuse in childhood are twice as likely to develop depression and/or anxiety disorders compared to others (14). Individuals with higher levels of childhood trauma have been found to have reduced grey matter volume in areas like the hippocampus and amygdala, possibly making them more sensitive to the negative emotions of others (15). Numerous studies have shown that emotional abuse is associated with increased risk of psychological distress, such as depression and anxiety symptoms, in adolescents (16), community adults (17), and clinical samples (18). Moreover, individuals who suffer from psychological distress are more likely to experience marital problems (19) and report marital dissatisfaction (20). Childhood emotional abuse (CEM) has also been found to be linked to marital dissatisfaction (21). Various factors might contribute to this association, including expression suppression strategies that affect romantic relationship satisfaction (22). Expression suppression is an emotion regulation strategy centered on suppressing ongoing emotional expressions (23). Gottman and Levenson (24) discovered that couples who display a higher ratio of positive to negative emotional behaviors during conflict interactions tend to have higher marital satisfaction, whereas those who maintain negative emotional states are more likely to face relationship difficulties (25). Therefore, Hypothesis 1 is that childhood trauma is significantly negatively correlated with romantic relationship satisfaction.

Childhood trauma refers to experiences of abuse or neglect during childhood, including emotional abuse, physical abuse, sexual abuse, emotional neglect, and physical neglect (26). Psychological distress resulting from childhood trauma increases the likelihood of experiencing marital problems and reporting marital dissatisfaction. Childhood trauma makes individuals more susceptible to negative emotions such as anxiety, depression, and anger. Studies indicate that maltreatment in childhood has long-term effects on mental health, with an increased risk of psychological distress for victims of emotional abuse in adolescence, adulthood, and clinical samples. Studies have found that adults with childhood trauma histories are more likely to encounter problems in romantic relationships (27, 28). Expression suppression strategies have been shown to mediate the impact of childhood trauma on romantic relationship satisfaction (22). Thus, Hypothesis 1: Childhood trauma is significantly negatively correlated with romantic relationship satisfaction.

Attachment refers to the connections formed in childhood. Under conditions of severe childhood adversity, these behavioral patterns may lead to dysfunctional behavior and cause challenges in adult relationships (29). According to Bowlby’s attachment theory, attachment relationships formed with caregivers in early childhood serve as the foundation for an individual’s internal working model, significantly affecting their future life and the underlying model of their marital relationships. This internal working model provides an internalized sense of security, allowing individuals to regulate emotions relatively autonomously and effectively. However, internalized early traumatic experiences can shape insecure attachment patterns and hinder emotional regulation. Past research has shown that childhood trauma negatively impacts parent-child relationships and contributes to insecure attachment styles (29), affecting psychological, physiological, and behavioral development. Trauma experienced during childhood enhances sensitivity in the behavioral activation system, making individuals overly dependent on attachment figures and susceptible to attachment anxiety (30). Collins and Reed (4) extended attachment theory and found that adult attachment styles predict relationship outcomes, particularly communication quality, trust, and overall satisfaction (31). Secure attachment types tend to have more positive feelings about their relationships, better communication, and greater trust, while anxious attachment types are characterized by fears of abandonment, insecurity in relationships, and lower levels of trust and satisfaction. Research conducted by Yun Li et al. (32) also shows that attachment type influences romantic relationship satisfaction, with secure attachment being associated with greater satisfaction. This could be attributed to more positive and proactive communication with partners among securely attached individuals. Therefore, Hypothesis 2: Attachment plays a mediating role in the relationship between childhood trauma and romantic relationship satisfaction.

Perceived social support is defined as an individual’s belief or expectation that their social network, including peers, family members, and significant others, will provide support when needed (33). Social support is closely linked to an individual’s overall psychological state (34). A strong social support system contributes to more positive emotional experiences and promotes psychological well-being, while a lack of social support from family, peers, and the community can have detrimental effects on mental health. According to Herman’s theory of childhood trauma, individuals with childhood trauma often remain in a state of helplessness and find it challenging to establish trust. These feelings and behaviors may extend into adult relationships, affecting social support levels (35). Despite the association between childhood trauma and reduced social support, strong social support has been suggested as a buffer against the negative effects of childhood trauma (36, 37). The stress-buffering model indicates that victims of maltreatment, including neglect, are more likely to downplay adversities or reevaluate stressors positively if they feel supported by a partner (38). The main effects model suggests that social support positively affects individuals, regardless of stress levels. Previous studies have confirmed that perceived social support positively predicts romantic relationship satisfaction (32). Attachment theory posits that internalized early trauma experiences create insecure attachment patterns, but social support can buffer against these negative effects (39). Therefore, Hypothesis 3: Perceived social support can moderate the impact of childhood trauma on romantic relationship satisfaction and also moderate the initial pathway of attachment.

**Figure 1:**
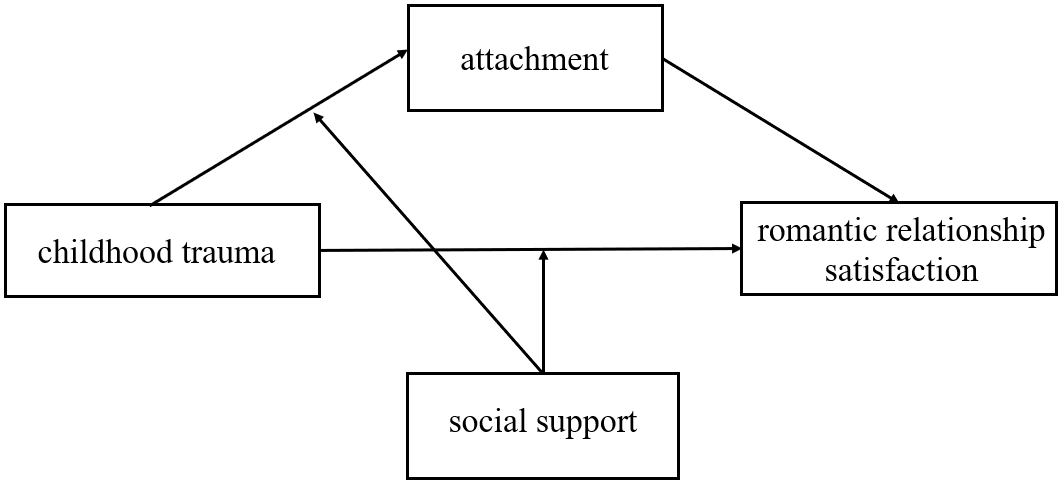
Moderated model.

In conclusion, this study aims to explore the relationships between childhood trauma, attachment, perceived social support, and romantic relationship satisfaction among college students, shedding light on the mechanisms through which childhood trauma impacts romantic relationship satisfaction. The hypothetical model is presented in **Figure 1**.

## 2 Methods

### 2.1 Participants

This study recruited 1,404 college students from six cities in Anhui and Hubei provinces, including Wuhu, Anqing, Chaohu, Bengbu, Fuyang, and Wuhan. Participants were drawn from various universities and academic disciplines, ensuring a diverse and representative sample. The majority were first- and second-year students, with 63.5% identifying as female, 32.1% as male, and 4.5% not reporting gender. Additionally, 66% of participants came from urban areas and 34% from rural areas. The average age was 23.86 years (SD = 1.20). The sample size was determined based on statistical requirements for factor analysis and regression analysis. Following MacCallum et al. (64), we used the guideline of 5–10 participants per item for factor analysis, resulting in a required sample size of 330–660 for 66 items. Additionally, Green’s (65) recommendation of 10–20 times the number of predictors for regression analysis further informed our calculations. The final dataset of 1,404 valid responses far exceeded these requirements, ensuring robust statistical power and reliable results for subsequent analyses.

A stratified random sampling method was employed. Participants were stratified by grade and gender to ensure balanced representation across these categories. Universities were randomly selected from six cities, encompassing a range of geographic and academic diversity. This approach ensured the sample was broadly representative of the target population of college students. Data were collected through an online survey distributed via the Questionnaire Star platform (https://www.wjx.cn/). College counselors shared the survey link in class WeChat and QQ groups, and students accessed the survey using a QR code or hyperlink. Incomplete or invalid responses were excluded, resulting in a final dataset of 1,404 valid responses.

The study was approved by the Ethics Review Committee of Anhui Normal University. All participants provided informed consent and were assured of their right to withdraw at any time. Personal data were anonymized, and all responses were kept strictly confidential, ensuring ethical compliance and privacy protection throughout the research process.

### 2.2 Measures

#### 2.2.1 Childhood trauma questionnaire - short form

The Chinese version of the Childhood Trauma Questionnaire, originally compiled by Bernstein and revised by domestic scholars (40), was used to measure childhood trauma among college students. This questionnaire includes five subscales (emotional abuse, physical abuse, sexual abuse, emotional neglect, and physical neglect) and contains 28 questions. Responses are rated on a five-point Likert scale ranging from “never” to “always.” Total scores range from mild to severe childhood trauma. In this study, the overall Cronbach’s alpha coefficient was 0.85.

#### 2.2.2 Adult attachment scale

The Adult Attachment Scale (AAS) was used to assess attachment, consisting of 18 items, divided into three subscales: closeness (6 items), dependence (6 items), and anxiety (6 items). Participants rate items on a five-point Likert scale (1 = “not characteristic of me” to 5 = “very characteristic of me”), with reverse scoring applied to certain items. In this study, the overall Cronbach’s alpha coefficient was 0.95.

#### 2.2.3 Perceived social support scale

The Perceived Social Support Scale (PSSS), developed by Blumenthal et al. in 1987 (66) and later revised by Jiang (67), was used to assess perceived social support. This scale includes 12 items measuring support from friends, family, and significant others, with responses rated on a seven-point Likert scale. The higher the total score, the greater the perceived social support. The Cronbach’s alpha coefficient for this study was 0.97.

#### 2.2.4 Romantic relationship satisfaction scale

The Intimate Relationship Satisfaction Scale, developed by Shen Liang (2005), was used to assess romantic relationship satisfaction. The scale includes seven items rated on a five-point scale, with higher scores indicating greater satisfaction with the relationship. The Cronbach’s alpha coefficient for this study was 0.77.

## 3 Results

### 3.1 Common method bias testing

SPSS 25.0 was used for analysis, along with the PROCESS macro developed by Andrew Hayes, to test the model. Harman’s single-factor test identified 11 factors with eigenvalues greater than 1, with the first factor explaining 23.31% of the variance, which is well below the 40% threshold (41), indicating no significant common method bias.

### 3.2 Descriptive statistics and correlation analysis

In this study, there were 891 female participants (66.4%) and 450 male participants (33.6%). The distribution by academic year was as follows: 608 first-year students (43.3%), 726 second-year students (51.7%), 58 third-year students (4.1%), and 12 fourth-year students (0.9%). A total of 886 students (66%) were from urban areas, while 456 students (34%) were from rural areas. Among the participants, 418 (31.1%) were only children, and 928 (68.9%) were non-only children.

The results, as shown in **Table 1**, indicate that childhood trauma was significantly negatively correlated with romantic relationship satisfaction (r = -0.06, *p* < 0.05) and significantly positively correlated with attachment (r = 0.09, *p* < 0.01). Attachment was significantly positively correlated with social support (r = 0.17, *p* < 0.001) and romantic relationship satisfaction (r = 0.14, *p* < 0.001). Additionally, social support was significantly positively correlated with romantic relationship satisfaction (r = 0.27, *p* < 0.001).

**Table 1:** Statistics and correlation analysis results (N = 1404).

Variables	M	SD	Childhood Trauma	Attachment	Social Support	Romantic Relationship Satisfaction
Childhood Trauma	61.37	8.48	1.00			
Attachment	54.69	5.42	0.09**	1.00		
Social Support	58.59	12.19	0.01	0.17***	1.00	
Romantic Relationship Satisfaction	24.17	5.21	-0.06*	0.14***	0.27***	1.00

p < 0.05, **p < 0.01, ***p < 0.001. The same applies hereinafter.

### 3.3 Gender differences in childhood trauma, attachment, social support, and romantic relationship satisfaction

To explore gender differences in childhood trauma, attachment, social support, and romantic relationship satisfaction among college students, independent sample t-tests were conducted. The results are presented in **Table 2**. The findings revealed significant gender differences in childhood trauma scores (*t*(1339) = 3.56, *p* < 0.001, Cohen’s d = 8.29), with male students scoring significantly higher than female students. Attachment also showed significant gender differences (*t*(1339) = 2.19, *p* = 0.029, Cohen’s d = 5.56), with male students reporting significantly higher attachment scores than female students. Social support displayed a significant gender difference (*t*(1339) = -5.64, *p* < 0.001, Cohen’s d = 12.12), with female students scoring significantly higher than male students. Finally, romantic relationship satisfaction showed significant gender differences (*t*(1339) = 3.14, *p* = 0.002, Cohen’s d = 5.17), with male students reporting significantly higher satisfaction levels than female students.

**Table 2:** Test of gender differences.

Variables	Male(n=450)	Female(n=89)	t	p	Cohen’s d
	M±SD	M±SD			
Childhood Trauma	62.74±10.84	60.76±6.64	3.56	<0.001	8.29
Attachment	55.30±5.83	54.57±5.41	2.19	0.029	5.56
Social Support	56.08±13.17	60.06±11.56	-5.64	<0.001	12.12
Romantic Relationship Satisfaction	24.80±5.40	23.82±5.06	3.14	0.002	5.17

### 3.4 Grade-level differences in childhood trauma, attachment, social support, and romantic relationship satisfaction

To investigate differences in childhood trauma, attachment, social support, and romantic relationship satisfaction among college students across different grade levels, a one-way ANOVA was conducted. The results are presented in **Table 3**. The analysis revealed significant differences in childhood trauma levels across grade levels (F(3, 1400) = 3.99, *p* = 0.008, η² = 0.01). First-year students reported significantly higher childhood trauma scores compared to second-year students. Attachment levels also showed significant differences among grade levels (F(3, 1400) = 9.47, *p* < 0.001, η² = 0.02), with first-year students scoring significantly higher than second-year students. Social support demonstrated significant grade-level differences (F(3, 1400) = 2.99, *p* = 0.030, η² = 0.01), with first-year students reporting significantly higher scores compared to second-year students. Romantic relationship satisfaction also differed significantly by grade level (F(3, 1400) = 16.20, *p* < 0.001, η² = 0.04). First-year students had significantly higher satisfaction levels than second-year students, and third-year students reported significantly higher satisfaction levels than second-year students.

**Table 3:** Test of grade differences.

Variables	M	SD	F(3,1400)	*p*	η^2^	
Childhood Trauma	61.50	8.41	3.99	0.008	0.01	freshman>sophomore
Attachment	54.80	5.54	9.47	<0.001	0.02	freshman>sophomore
Social Support	58.66	12.21	2.99	0.030	0.01	freshman>sophomore
Romantic Relationship Satisfaction	24.11	5.18	16.20	<0.001	0.04	freshman>sophomore, junior>sophomore

### 3.5 The relationship between college students’ childhood trauma experiences and romantic relationship satisfaction: a moderated mediation model test

To explore the relationships among the main continuous variables, Pearson’s coefficients were used to obtain bivariate correlations. Next, based on the results of the correlation analysis, in order to test the moderated mediation effect, the independent variable (childhood trauma), the mediating variable (attachment), the moderating variable (social support), and the dependent variable (romantic relationship satisfaction) were standardized into Z-scores. To control for potential confounding factors, demographic variables, including gender, age, only-child status, and grade level, were included as covariates in the analysis. Following the steps for testing moderated mediation effects, the PROCESS macro for SPSS, developed by Hayes, was employed. This analysis sequentially tested the roles of social support and attachment in the relationship between childhood trauma and romantic relationship satisfaction.

The results, as shown in **Table 4**, indicated that childhood trauma significantly negatively predicted romantic relationship satisfaction (*β* = -0.06, *t* = -2.11, *p* < 0.05). This negative effect persisted even when the mediator (attachment) was included in the model (*β* = -0.07, *t* = -2.59, *p* < 0.05). Childhood trauma significantly predicted attachment (*β* = 0.09, *t* = 3.20, *p* < 0.001), and attachment significantly predicted romantic relationship satisfaction (*β* = 0.14, *t* = 5.05, *p* < 0.001).

**Table 4:** Test of mediating effect of attachment.

Regression equation(N=1404)		Fit index			Significance of correlation coefficient		
Result variable	Predictor variable	R^2^	*F*	*β*	BootCI Lower Limit	BootCI Upper Limit	t
Romantic Relationship Satisfaction		0.04	10.81***				
	Gender			-0.07	-0.27	-0.02	-2.26*
	Age			0.15	0.07	0.18	4.67***
	Being an Only Child			0.02	-0.07	0.18	0.82
	Grade			-0.19	-0.43	-0.21	-5.70***
	Childhood Trauma			-0.06	-0.12	-0.01	-2.11*
Attachment		0.04	9.78				
	Gender			-0.01	-0.14	0.10	-0.28
	Age			0.16	0.08	0.18	4.88***
	Being an Only Child			-0.02	-0.16	0.09	-0.56
	Grade			-0.15	-0.36	-0.15	-4.68***
	Childhood Trauma			0.09	0.03	0.14	3.20***
Romantic Relationship Satisfaction		0.06	13.43				
	Gender			-0.07	-0.26	-0.02	-2.24*
	Age			0.13	0.05	0.16	3.97***
	Being an Only Child			0.03	-0.07	0.18	0.91
	Grade			-0.16	-0.39	-0.17	-5.03***
	Childhood Trauma			-0.07	-0.13	-0.02	-2.59*
	Attachment			0.14	0.09	0.21	5.05***

A moderated mediation analysis was conducted using PROCESS Model 8 developed by Hayes. The results, as shown in **Table 5**, revealed that when social support was included in the model, childhood trauma positively predicted attachment (*β* = 0.15, *t* = 4.43, *p* < 0.001), and social support positively predicted attachment (*β* = 0.15, *t* = 5.45, *p* < 0.001). The interaction term between childhood trauma and social support also significantly predicted attachment (*β* = 0.09, *t* = 3.18, *p* < 0.001). However, social support did not moderate the direct effect of childhood trauma on romantic relationship satisfaction.

**Table 5:** Test of moderated mediation effect.

Regression equation(N=1404)		Fit index			Significance of correlation coefficient		
Result variable	Predictor variable	R^2^	*F*	*β*	BootCI Lower Limit	BootCI Upper Limit	t
Attachment		0.13	21.34***				
	Gender			-0.08	-0.20	0.04	-1.25
	Age			0.13	0.07	0.18	4.83***
	Being an Only Child			-0.04	-0.16	0.08	-0.60
	Grade			-0.23	-0.33	-0.12	-4.23***
	Childhood Trauma			0.15	0.08	0.21	4.43***
	Social Support			0.15	0.10	0.21	5.45***
	Childhood Trauma × Social Support			0.09	0.03	0.14	3.18**
Romantic Relationship Satisfaction		0.13	21.34				
	Gender			-0.24	-0.36	-0.12	-3.90***
	Age			0.11	0.60	0.16	4.22***
	Being an Only Child			0.06	-0.06	0.18	0.10
	Grade			-0.25	-0.36	-0.15	-4.70***
	Childhood Trauma			-0.06	-0.13	0.01	-1.86
	Attachment			0.10	0.05	0.16	3.58***
	Social Support			0.26	0.20	0.31	9.03***
	Childhood Trauma × Social Support			0.01	-0.04	0.07	0.52

The results from **Table 6** indicate that a suppression effect was observed in the mediation analysis. The direct effect of childhood trauma on romantic relationship satisfaction was significant (*β* = -0.07, 95% CI = [-0.13, -0.02]), while the indirect effect through attachment was also significant (β = 0.01, 95% CI = [0.01, 0.02]). The total effect was estimated at *β* = -0.06, with the direct effect accounting for 116.67% and the indirect effect contributing 16.67%.

**Table 6:** Mediation effect analysis.

	Effect Size	Boot SE	Bootci Lower Limit	BootCI Upper Limit	Relative Effect Value
Direct effect	–0.07	0.03	–0.13	–0.02	116.67%
Mediating effect of Attachment	0.01	0.01	0.01	0.02	16.67%
Total effect	–0.06	0.03	–0.12	-0.004	

**Figure 2:**
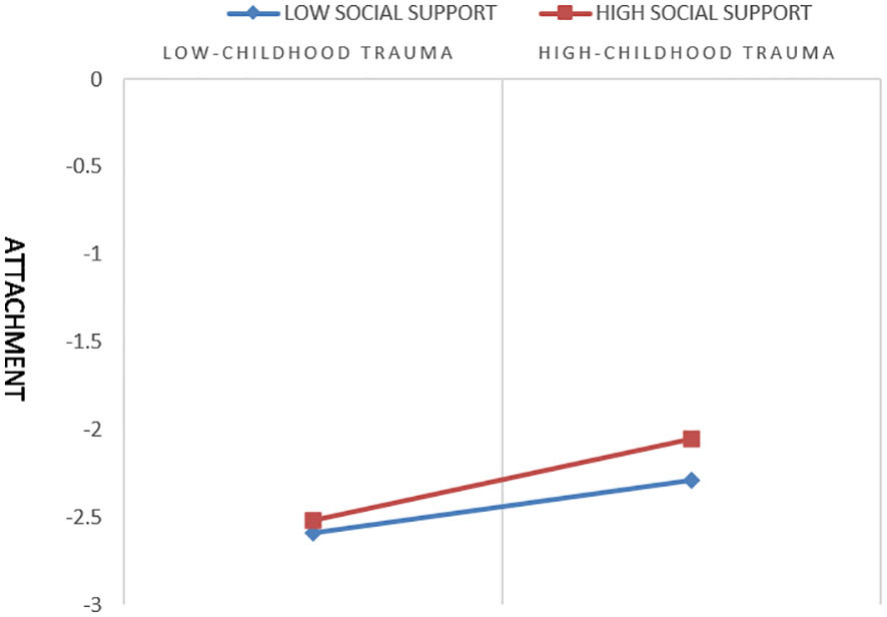
Moderated mediation.

To further examine whether social support significantly moderated the relationship between childhood trauma and romantic relationship satisfaction, simple slope analysis was conducted by categorizing social support into high and low groups (one standard deviation above and below the mean, respectively). As shown in **Figure 2**, when social support was low (one standard deviation below the mean), childhood trauma significantly predicted attachment (*simple slope* = 0.06, *t* = 2.18, *p* = 0.03). Similarly, when social support was high (one standard deviation above the mean), childhood trauma also significantly predicted attachment (*simple slope* = 0.23, *t* = 4.37, *p* < 0.001). These results suggest that the positive predictive effect of childhood trauma on attachment becomes stronger as the level of social support increases.

## 4 Discussion

This study demonstrates that childhood trauma negatively predicts romantic relationship satisfaction both directly and indirectly through attachment. Social support moderates the relationship between childhood trauma and attachment, but it does not moderate the direct effect of childhood trauma on romantic relationship satisfaction. These findings highlight the mediating role of attachment and the moderating role of social support in the context of childhood trauma and romantic relationships.

### 4.1 The relationship between childhood trauma and romantic relationship satisfaction

This study examined the relationships among childhood trauma, attachment, social support, and romantic relationship satisfaction. After controlling for grade, it was found that childhood trauma negatively predicted romantic relationship satisfaction, which is consistent with previous research. Cohen et al. (42) found that childhood trauma weakens an individual’s ability to establish and maintain intimate relationships with others. Ji et al. (43, 44) indicated that childhood trauma significantly positively predicts fear of intimacy. According to Bowen’s family systems theory, the original family can shape an individual’s perception of intimate relationships, with motivations behind people’s behaviors rooted in interactions and experiences gained within the family during their upbringing (45). Therefore, trauma suffered during childhood, such as physical neglect or emotional neglect, can lead to low levels of self-differentiation, greatly affecting the individual’s behavior in future intimate relationships and increasing the likelihood of problematic intimate relationships. Additionally, social learning theory suggests that behaviors are acquired by observing others, so individuals who have experienced childhood trauma are also likely to treat their partners with violence, neglect, or indifference, thereby damaging the intimacy of the relationship (46, 47).

### 4.2 The mediating role of attachment

This study also found that attachment partially mediates the relationship between childhood trauma and romantic relationship satisfaction. Childhood trauma not only directly predicts romantic relationship satisfaction but also indirectly predicts college students’ romantic relationship satisfaction through attachment, which aligns with our hypothesis. According to the internal working model of attachment, the attachment relationship formed between a child and their caregiver in early childhood influences their subsequent life and serves as the fundamental working model in future intimate relationships. Increasing evidence shows that adverse childhood experiences disrupt a child’s sense of security, which is the foundation for the development of the attachment working model (48, 49). Studies have confirmed that attachment is closely related to early experiences, with negative early life experiences linked to the development of insecure attachment (50, 51). Attachment plays a crucial role in intimate relationships—it can be understood as a complex pattern of interpersonal interactions related to security, emotional regulation, coping with stress, and pain. Individuals with secure attachment exhibit more positive psychological behaviors and have higher romantic relationship satisfaction compared to those with insecure attachment (52). Previous studies have also confirmed that attachment directly predicts romantic relationship satisfaction among college students (32, 53).

### 4.3 The moderating role of social support

This study also found that social support moderates the predictive effect of childhood trauma on attachment, and the positive predictive effect of childhood trauma on attachment strengthens as social support increases. Herman’s childhood trauma theory suggests that individuals with childhood trauma are prone to developing negative self-concepts, which can lead to negative emotions and a state of helplessness, making it difficult for them to establish trust with others. These feelings and behaviors may persist into adulthood, affecting social support levels. Close social relationships and secure attachment are believed to have stress-relieving functions, providing a sense of safety and promoting psychological well-being (54, 55). Conversely, insecure attachment may lead to poorer psychological well-being. Although childhood trauma is related to lower social support, good social support is a mechanism that can buffer the negative effects of childhood trauma. Studies have shown that social support can offset the harmful consequences of abuse (56). For example, studies on school-age children and adolescents have found that abused individuals with high social support are less likely to experience symptoms of depression and anxiety (57) or engage in risky or violent behaviors (58–60). Good social support plays an intervening role in mitigating the negative effects of early life stress, brings positive psychological factors, and may reduce the occurrence of early life stress (61, 62). It is noteworthy that the results of this study indicate that social support does not significantly moderate the direct relationship between childhood trauma and romantic relationship satisfaction, possibly because social support alone may not be sufficient to overcome the harmful effects of abuse (63).

### 4.4 Implications and limitations

This study provides valuable insights into the relationships between childhood trauma, attachment, social support, and romantic relationship satisfaction, offering new perspectives for both theoretical understanding and practical applications. It reveals that childhood trauma negatively predicts romantic relationship satisfaction both directly and indirectly through attachment. Moreover, social support moderates the relationship between childhood trauma and attachment, though it does not directly moderate the influence of childhood trauma on romantic relationship satisfaction. These findings contribute to our theoretical understanding of how early trauma impacts romantic relationships in adulthood.

Beyond its theoretical contributions, the findings have important practical implications for supporting university students. Emotional distress stemming from childhood trauma and toxic relationships can affect not only students’ personal lives but also their academic performance and social relationships. Students with unresolved emotional issues or insecure attachment may face challenges with concentration, motivation, and time management, which could hinder academic achievement. Furthermore, difficulties in romantic relationships may spill over into broader social contexts, negatively impacting peer interactions and participation in group activities.

University counselors and mental health services are uniquely equipped to address these challenges by offering targeted support programs. Counselors can assist students in recognizing the signs of unhealthy relationships and developing effective strategies to manage emotional difficulties. Interventions such as emotional education, establishing emotional boundaries, and preventive programs could help mitigate the psychological impact of trauma and promote mental well-being. Additionally, incorporating social support initiatives into counseling services could strengthen students’ resilience, enhancing both their academic performance and social experiences.

While this study provides significant theoretical and empirical insights, it has certain limitations. First, the cross-sectional design limits the ability to establish causal relationships between the variables. Although significant correlations were observed among childhood trauma, attachment, and romantic relationship satisfaction, the causal direction remains uncertain. Future research could benefit from employing longitudinal designs to better understand the temporal dynamics and causal pathways of these relationships.

Second, this study primarily focused on the overall dimensions of each questionnaire. The attachment questionnaire used in this research includes three distinct dimensions: secure, anxious, and avoidant attachment. Prior studies indicate that individuals with different attachment styles exhibit varying behaviors and emotional responses in intimate relationships. Therefore, future research could delve into the specific roles of these attachment dimensions in shaping romantic relationship satisfaction and related psychological outcomes.

Additionally, the study did not address the trajectory of unsatisfactory relationships, particularly how they might progress to consensual breakups or escalate into violent or aggressive separations. This is a critical area for understanding the influence of early trauma on romantic dissatisfaction. Relationship satisfaction often declines in a nonlinear manner, marked by conflicts, emotional dysregulation, and communication breakdowns, which may be exacerbated by early trauma. Understanding these hidden dynamics is essential for uncovering how relationship dissatisfaction evolves and intensifies.

Future research should investigate how childhood trauma, particularly in both men and women, impacts romantic relationship satisfaction and may lead to more severe outcomes such as violent breakups. Areas of focus could include emotional regulation, attachment patterns, and conflict resolution strategies as mediators that exacerbate relational dissatisfaction. These insights could inform interventions designed to address relationship distress stemming from trauma and foster healthier relational dynamics.

Furthermore, future studies should aim to refine the concepts of “unsatisfactory relationship” and “satisfactory relationship” to better capture their dynamic complexities. This includes exploring underlying factors such as conflict, emotional dysregulation, and aggression, which may contribute to satisfaction or dissatisfaction levels. A more nuanced understanding of these terms could help uncover the intricate realities within romantic relationships, providing a stronger foundation for targeted interventions and support programs.

## 5 Conclusion

(1) Childhood trauma in college students is significantly positively correlated with attachment and significantly negatively correlated with romantic relationship satisfaction. Attachment is significantly positively correlated with both social support and romantic relationship satisfaction, and social support is significantly positively correlated with attachment and romantic relationship satisfaction.
(2) Childhood trauma affects romantic relationship satisfaction through the indirect effect of attachment.
(3) Social support can moderate the relationship between childhood trauma and attachment. The higher the level of social support, the stronger the predictive effect of childhood trauma on attachment.
(4) Future research should investigate how childhood trauma impacts romantic relationship satisfaction and its potential to result in severe outcomes, such as violent breakups. These insights could lay the groundwork for interventions targeting relationship distress, fostering healthier relational dynamics. Additionally, future interventions should consider the broader effects of toxic relationships, addressing not only emotional well-being but also academic performance and social interactions, to promote more positive outcomes for university students.

## Data Availability

Requests to access these datasets should be directed to lijuanq@ahnu.edu.cn.
