# Supplementary material for: The relationship between childhood trauma and romantic relationship satisfaction: the role of attachment and social support
Source: Front Psychiatry. 2025 Jan 22;15:1519699. doi: 10.3389/fpsyt.2024.1519699 (PMC11795211; doi:10.3389/fpsyt.2024.1519699)
Supplement: Supplementary file 1 [file DataSheet1.pdf]

调查问卷

请填写你的基本情况：

1. 性别：① 男　　② 女

2. 出生年份：________（年）

3. 是否独生子女：① 是　　② 否

4.你现在所处的年级：①大一　②大二　③大三　④大四

5. 是否有过留守经历（16 岁前，你的父母双方或者一方外出工作或打工，持续离家 6 个月或更长）：① 是　　② 否

6. 你父母的婚姻状况：①初婚　②离异　③再婚　④其他

7.你的家庭月收入为：①2000 以下　②2000—4000　③4000 以上

8.你的家庭住址：①大城市或者省会城市　②中等城市　③小城市　④县城　⑤农村

9.你的父亲教育水平：①小学及小学以下 ②初中 ③高中（含中专、技校、职高） ④大专 ⑤本科 ⑥硕士 ⑦博士

10.你的母亲教育水平：①小学及小学以下 ②初中 ③高中（含中专、技校、职高） ④大专 ⑤本科 ⑥硕士 ⑦博士

A.本问卷调查的是你儿童期（16 岁以前）的成长经历。请根据你**当时的**体会从五个选项中**圈出**（只选一项）最适合你情况的答案。

1=从来没有这种现象　2=偶尔　3=有时　4=经常　5=总是

	题目	从来没有	偶尔	有时	经常	总是
1	当时家里没人关心我的饥饱	1	2	3	4	5
2	当时有人照顾我、保护我	1	2	3	4	5
3	当时家里有人喊我“笨蛋”、“懒虫”或“丑八怪”等	1	2	3	4	5
4	当时我的父母因为酗酒、吸毒或者赌博而不能照顾家庭	1	2	3	4	5
5	当时家里有人重视我	1	2	3	4	5
6	当时家里没人管我衣着冷暖	1	2	3	4	5
7	当时我感到家里人爱我	1	2	3	4	5
8	当时我觉得父母希望从来没有生过我	1	2	3	4	5
9	当时家里有人把我打伤的很重，不得不去医院	1	2	3	4	5
10	当时我家的状况需要改善	1	2	3	4	5
11	当时家里有人打得我皮肤青紫或留下伤痕	1	2	3	4	5
12	当时家里有人用皮带、绳子、木板或其它硬东西惩罚我	1	2	3	4	5
13	当时家里人彼此互相关心	1	2	3	4	5
14	当时家里有人向我说过侮辱性或让我伤心的话	1	2	3	4	5
15	我当时受到了躯体虐待	1	2	3	4	5
16	我觉得我的童年比任何人的都完美	1	2	3	4	5
17	当时我被打得很重，引起了老师、邻居或医生等人的注意	1	2	3	4	5
18	当时我觉得家里有人恨我	1	2	3	4	5
19	当时家里人关系很亲密	1	2	3	4	5
20	当时有人以带有性色彩的方式触摸我或让我触摸他/她	1	2	3	4	5
21	当时有人威胁我让我同他/她做性方面的事	1	2	3	4	5
22	我觉得我的家好得不能再好了	1	2	3	4	5
23	当时有人试图让我做或看性方面的事	1	2	3	4	5
24	当时有人猥亵我、如要流氓、动手动脚等	1	2	3	4	5
25	当时我的心灵受到了折磨或虐待	1	2	3	4	5
26	当时有人关心我的身体健康	1	2	3	4	5
27	当时我受到了性虐待	1	2	3	4	5
28	当时家是我获得力量和支持的源泉	1	2	3	4	5
29	在你的一生中，第一次性行为时你多大年龄？<br>（如从来没有，填“88”）					

B.请阅读下列语句，考虑你的**所有关系**（过去的和现在的），并回答以下题目，如果你从来没有卷入进情感关系中，请按你认为的情感会是怎样的来回答。**圈出**（只选一项）最能表达你的感受的数字。

1=完全不符合　2=比较不符合　3=不能确定　4=比较符合　5=完全符合。

	**题目**	**完全不符合**	**比较不符合**	**不能确定**	**比较符合**	**完全符合**
1	我发现与人亲近比较容易	1	2	3	4	5
2	我发现要我去依赖别人很困难	1	2	3	4	5
3	我时常担心情侣并不真心爱我	1	2	3	4	5
4	我发现别人并不愿像我希望的那样亲近我	1	2	3	4	5
5	能依赖别人让我感到很舒服	1	2	3	4	5
6	我不在乎别人太亲近我	1	2	3	4	5
7	我发现当我需要别人帮助时，没人会帮我	1	2	3	4	5
8	和别人亲近使我感到不舒服	1	2	3	4	5
9	我时常担心伴侣不想和我在一起	1	2	3	4	5
10	当我对别人表达我的情感时，我害怕他们会与我的感觉不一样	1	2	3	4	5
11	我时常怀疑情侣是否真正关心我	1	2	3	4	5
12	我对别人建立亲密关系感到舒服	1	2	3	4	5
13	当有人在情感上太亲近我时，我感到不舒服	1	2	3	4	5
14	我知道当我需要别人帮助时，总会有人帮我	1	2	3	4	5
15	我想与人亲近，但担心自己会受伤害	1	2	3	4	5
16	我发现我很难完全信赖别人	1	2	3	4	5
17	情侣想要我在情感上更亲近一些，这常使我感到不舒服	1	2	3	4	5
18	我不能肯定，在我需要时，总找得到可以依赖的人	1	2	3	4	5

C.以下 12 个句子，每一个句子后各有 7 个答案。请你根据自己的实际情况在每句后面**圈出**（只选一项）最能描述你的**实际情况**的一句。

1=极不同意　2=很不同意　3=稍不同意　4=中立　5=稍同意　6=很同意　7=极同意

	**题目**	**极不同意**	**很不同意**	**稍不同意**	**中立**	**稍同意**	**很同意**	**极为同意**
1	在我遇到问题时有些人（领导、亲戚、同事）会出现在我的身旁	1	2	3	4	5	6	7
2	我能够与有些人（领导、亲戚、同事）共享快乐与忧伤	1	2	3	4	5	6	7
3	我的家庭能够切实具体地给我帮助	1	2	3	4	5	6	7
4	在需要时我能够从家庭获得情感上的帮助和支持	1	2	3	4	5	6	7
5	当我有困难时有些人（领导、亲戚、同事）是安慰我的真正源泉	1	2	3	4	5	6	7
6	我的朋友们能真正的帮助我	1	2	3	4	5	6	7
7	在发生困难时，我可以依靠我的朋友们	1	2	3	4	5	6	7
8	我能与自己的家庭谈论我的难题	1	2	3	4	5	6	7
9	我的朋友们能与我分享快乐与忧伤	1	2	3	4	5	6	7
10	在我的生活中有些人（领导、亲戚、同事）关心着我的感情	1	2	3	4	5	6	7
11	我的家庭能心甘情愿协助我作出各种决定	1	2	3	4	5	6	7
12	我能与朋友们讨论自己的难题	1	2	3	4	5	6	7

D.这是一份了解你目前**恋爱满意度**的问卷，请你仔细阅读每个句子，根据你的实际情况，**圈出**（只选一项）你对句子中描述内容的符合程度。

1=完全不符合　2=有点不符合　3=一般　4=有点符合　5=完全符合

	**题目**	**完全不符合**	**有点不符合**	**一般**	**有点符合**	**完全符合**
1	我把对方当做自己的亲人	1	2	3	4	5
2	我总是十分信任对方	1	2	3	4	5
3	与其他任何事情相比，这种恋爱关系给我的快乐最多	1	2	3	4	5
4	我们的关系会发展的很好	1	2	3	4	5
5	我后悔选择对方	1	2	3	4	5
6	我没有从对方那里感受过应有的爱	1	2	3	4	5
7	我和对方的生活哲学迥然不同	1	2	3	4	5
