# Supplementary material for: The relationship between childhood trauma and romantic relationship satisfaction: the role of attachment and social support
Source: Front Psychiatry. 2025 Jan 22;15:1519699. doi: 10.3389/fpsyt.2024.1519699 (PMC11795211; doi:10.3389/fpsyt.2024.1519699)
Supplement: Supplementary file 2 [file DataSheet2.pdf]

Survey Questionnaire

Basic Information:

1. Gender:

   ① Male ② Female

2. Year of Birth:

   _______ (Year)

3. Are you an only child?

   ① Yes ② No

4. Current academic year:

   ① Freshman ② Sophomore ③ Junior ④ Senior

5. Have you experienced being left behind as a child (before age 16, when one or both parents left home for work for 6 months or more)?

   ① Yes ② No

6. Your parents' marital status:

   ① First marriage ② Divorced ③ Remarried ④ Other

7. Monthly household income:

   ① Below 2000 ② 2000–4000 ③ Above 4000

8. Home location:

   ① Major city or provincial capital ② Medium-sized city ③ Small city ④ County town ⑤ Rural area

9. Father's education level:

   ① Primary school or below ② Middle school ③ High school (including vocational, technical school, or secondary school)

   ④ Associate degree ⑤ Bachelor's degree ⑥ Master's degree ⑦ Doctorate

10. Mother's education level:

    ① Primary school or below ② Middle school ③ High school (including vocational, technical school, or secondary school)

    ④ Associate degree ⑤ Bachelor's degree ⑥ Master's degree ⑦ Doctorate

A. This questionnaire assesses your childhood experiences (before the age of 16). Please select the response that best reflects your experience at that time using the following scale:
1 = Never true; 2 = Rarely true; 3 = Sometimes true; 4 = Often true; 5 = Always true

	Item	Never true	Rarely true	Sometimes true	Often true	Always true
1	At that time, no one in my family cared about my hunger or fullness.	1	2	3	4	5
2	At that time, someone took care of me and protected me.	1	2	3	4	5
3	At that time, someone in my family called me "stupid," "lazy," or "ugly."	1	2	3	4	5
4	At that time, my parents couldn't take care of the family due to alcohol, drug abuse, or gambling.	1	2	3	4	5
5	At that time, someone in my family valued me.	1	2	3	4	5
6	At that time, no one in my family cared about my clothing or warmth.	1	2	3	4	5
7	At that time, I felt that my family loved me.	1	2	3	4	5
8	At that time, I felt that my parents wished they had never had me.	1	2	3	4	5
9	At that time, someone in my family hurt me so badly that I had to go to the hospital.	1	2	3	4	5
10	At that time, the situation in my family needed improvement.	1	2	3	4	5
11	At that time, someone in my family hit me so hard that I had bruises or scars.	1	2	3	4	5
12	At that time, someone in my family punished me with a belt, rope, board, or other hard objects.	1	2	3	4	5
13	At that time, people in my family cared for each other.	1	2	3	4	5
14	At that time, someone in my family said insulting or hurtful things to me.	1	2	3	4	5
15	At that time, I was physically abused.	1	2	3	4	5
16	At that time, I felt my childhood was better than anyone else's.	1	2	3	4	5
17	At that time, I was beaten so badly that it attracted the attention of teachers, neighbors, or doctors.	1	2	3	4	5
18	At that time, I felt that someone in my family hated me.	1	2	3	4	5
19	At that time, people in my family had a close relationship with each other.	1	2	3	4	5
20	At that time, someone touched me or made me touch them in a sexually suggestive way.	1	2	3	4	5
21	At that time, someone threatened me to do sexual things with them.	1	2	3	4	5
22	At that time, I felt my family was perfect.	1	2	3	4	5
23	At that time, someone tried to make me do or watch sexual acts.	1	2	3	4	5
24	At that time, someone molested me, such as groping or inappropriate physical contact.	1	2	3	4	5
25	At that time, my spirit was tortured or abused.	1	2	3	4	5
26	At that time, someone cared about my physical health.	1	2	3	4	5
27	At that time, I was sexually abused.	1	2	3	4	5
28	At that time, my family was a source of strength and support for me.	1	2	3	4	5

B. Please read the following statements and consider all your relationships (past and present). Answer the following questions. If you have never been involved in an emotional relationship, answer based on how you think an emotional relationship would be. Circle (select one) the number that best expresses your feelings.

1 = Strongly Disagree; 2 = Disagree; 3 = Neutral; 4 = Agree; 5 = Strongly Agree

	Item	Strongly Disagree	Disagree	Neutral	Agree	Strongly Agre
1	I find it easy to get close to others.	1	2	3	4	5
2	I find it difficult to rely on others.	1	2	3	4	5
3	I often worry that my partner does not truly love me.	1	2	3	4	5
4	I find that others are not as close to me as I would like them to be.	1	2	3	4	5
5	I feel comfortable relying on others.	1	2	3	4	5
6	I don’t mind others being too close to me.	1	2	3	4	5
7	I find that when I need help, no one is there to help me.	1	2	3	4	5
8	Getting close to others makes me feel uncomfortable.	1	2	3	4	5
9	I often worry that my partner doesn’t want to be with me.	1	2	3	4	5
10	When I express my feelings to others, I am afraid they may feel differently from me.	1	2	3	4	5
11	I often doubt whether my partner truly cares about me.	1	2	3	4	5
12	I feel comfortable forming close relationships with others.	1	2	3	4	5
13	When someone gets too emotionally close to me, I feel uncomfortable.	1	2	3	4	5
14	I know that when I need help, there will always be someone to help me.	1	2	3	4	5
15	I want to get close to others, but I am afraid of getting hurt.	1	2	3	4	5
16	I find it hard to fully trust others.	1	2	3	4	5
17	My partner wants me to be more emotionally close, and this often makes me uncomfortable.	1	2	3	4	5
18	I am not sure if there is always someone I can rely on when I need them.	1	2	3	4	5

C. The following 12 sentences each have 7 response options. Please select (only one) the answer that best describes your actual situation after each sentence.
1 = Strongly Disagree; 2 = Disagree; 3 = Slightly Disagree; 4 = Neutral; 5 = Slightly Agree;
6 = Agree; 7 = Strongly Agree

	Item							
1	Some people (e.g., leaders, relatives, colleagues) are there for me when I face problems.	1	2	3	4	5	6	7
2	I can share both my joys and sorrows with some people (e.g., leaders, relatives, colleagues).	1	2	3	4	5	6	7
3	My family is able to provide practical and concrete help to me.	1	2	3	4	5	6	7
4	I can receive emotional help and support from my family when needed.	1	2	3	4	5	6	7
5	Some people (e.g., leaders, relatives, colleagues) comfort me when I am facing difficulties.	1	2	3	4	5	6	7
6	My friends can truly help me.	1	2	3	4	5	6	7
7	In times of difficulty, I can rely on my friends.	1	2	3	4	5	6	7
8	I can discuss my problems with my family.	1	2	3	4	5	6	7
9	My friends can share both my joys and sorrows with me.	1	2	3	4	5	6	7
10	Some people (e.g., leaders, relatives, colleagues) care about my feelings in my life.	1	2	3	4	5	6	7
11	My family is willing to help me make various decisions.	1	2	3	4	5	6	7
12	I can discuss my problems with my friends.	1	2	3	4	5	6	7

D. This is a questionnaire to assess your current romantic relationship satisfaction. Please carefully read each statement and circle (select one) the degree to which the statement describes your situation.
1 = Strongly Disagree; 2 = Disagree; 3 = Neutral; 4 = Agree; 5 = Strongly Agree

	Item	Strongly Disagree	Disagree	Neutral	Agree	Strongly Agre
1	I consider the other person as my family.	1	2	3	4	5
2	I always trust the other person completely.	1	2	3	4	5
3	Compared to anything else, this romantic relationship brings me the most happiness.	1	2	3	4	5
4	Our relationship will develop well.	1	2	3	4	5
5	I regret choosing the other person.	1	2	3	4	5
6	I have not felt the love I deserve from the other person.	1	2	3	4	5
7	My life philosophy is completely different from the other person's.	1	2	3	4	5
